# Personalised surveillance after treatment for high-risk cancer

**DOI:** 10.18632/oncotarget.26619

**Published:** 2019-01-22

**Authors:** Christina Guo, Jeremy Lewin, Mark Shackleton

**Affiliations:** Mark Shackleton: Cancer Treatment and Development Laboratory, Central Clinical School, Monash University, Victoria, Australia; Department of Oncology, Alfred Health, Victoria, Australia

**Keywords:** cancer, melanoma, PET, ctDNA, surveillance

An important aim of imaging surveillance after cancer treatment is to identify asymptomatic relapse in the hope of maximising therapeutic salvage options that improve key clinical outcomes such as overall survival. Such surveillance is predicated on the assumption that early detection of relapse provides opportunity for potentially curative surgical resection and/or allows delivery of systemic therapies that may be more effective in the context of low-volume disease.

The notion that this may be beneficial is largely supported by retrospective case series, wherein resection of oligometastatic disease was associated with prolonged overall survival [1, 2]. Similarly, for systemic therapies, improved response to immunotherapy and targeted therapies may be associated with low-volume disease contexts facilitated by early detection [3, 4]. Nevertheless, imaging surveillance after treatment is not without risks and the potential benefits of early detection are not ubiquitous across tumour types [5]. With recent advances in cancer diagnostics and anti-cancer therapy, the risk-benefit ratio of post-treatment imaging surveillance is in flux.

Potential benefits of such surveillance need to be considered in context of the unwanted consequences of imaging, such as false positive findings, patient ‘scanxiety’, cumulative radiation exposure and cost. The rate of false positive findings during surveillance varies according to the pre-test probability of recurrence and the specificity of the surveillance modality used. As high sensitivity is a key requirement of imaging surveillance, this is often at the cost of reduced specificity and false positives.

In our previous report of sub-stage specific surveillance in stage 3 melanoma, ‘false positive’ incidental findings were found in 23% of patients, although most of these were due to benign processes. Further, potentially adverse consequences of our false positives were offset by the fact that many of them (6% of all patients in the surveillance cohort) were asymptomatic non-melanoma malignancies that were treated with curative intent [6]. False positive results of surveillance imaging are thus not always detrimental, although they are often a source of major patient anxiety. A survey of 103 North American patients with non-small cell lung cancer found that 83% experienced scan-associated distress, which led to impaired quality of life [7]. Furthermore, radiation exposure from imaging surveillance may be of concern in younger patient cohorts. For this reason, imaging surveillance after treatment for testicular cancer, which typically affects younger men, has trended towards less frequent imaging that is tailored to the temporal and spatial patterns of disease recurrence [8].

In light of concerns around imaging surveillance, novel approaches are being investigated. For example, the presence of circulating tumour DNA after curative-intent treatment has been shown to predict recurrence in early stage colorectal [9] and breast cancers [10]. Similarly, circulating microRNA has been shown to be predictive of residual germ cell tumour post chemotherapy [11]. As non-invasive tools appear sensitive for predicting risk of relapse, they are subjects of intense research and commercial interest that will likely have ramifications for future surveillance strategies. It is expected that combined imaging and non-invasive surveillance strategies will be developed as the roles become better defined for using these novel approaches for detecting cancer recurrence.

Regardless of the modalities of detection of relapsed disease, a key aspect of surveillance will remain its scheduling, which foremost must consider the pre-test probability of a true positive result at each potential surveillance time point. This will vary depending on relapse kinetics unique to each cancer type. For almost all cancers, however, the pre-test probability of a true positive result varies predominantly according to disease stage and the time after potentially curative treatment; latter stage cancers are more likely to recur than earlier stage cancers, and recurrence rates are typically higher within the first 2-3 years after initial treatment than those beyond 5 years. Accordingly, and to maximize the chance of detecting asymptomatic recurrence, more frequent monitoring might be necessary in the first 2-3 years after treatment, compared to beyond 5 years, and (for example) patients with TNM stage 3C cancers might be more frequently surveyed than those with stage 3A cancers, such as we recommend for melanoma [6].

A ‘one-size-fits-all’ approach to surveillance that ignores these nuances would be predicted to increase both false positive rates (usually from over-surveillance in low risk relapse contexts) and also the proportion of patients whose relapsed disease becomes symptomatic prior to the next planned surveillance time point (e.g. as a result of under-surveillance). We urge patient advocacy groups, medical practitioners, and funders of health care to collaborate towards agreement on pre-test probabilities of true positive findings of cancer relapse that justify the effort, expense, and patient anxiety of surveillance across malignant diseases in which early detection of relapse has the potential to benefit patients with effective salvage therapies.

We also encourage careful and prospective collection of outcome data within established surveillance programmes. Historically, studies that randomize patients to different surveillance approaches and schedules have been difficult to conduct. Complicating this, the data generated may become obsolete in rapidly changing therapy and technological landscapes. Suggested ways to address these issues include integrating evaluation of surveillance outcomes into existing clinical trials of adjuvant therapy or using registry-based studies [12]. Moreover, evaluation of surveillance should include economic modelling that accounts not only for the costs of surveillance but also economic benefits from early detection of improved patient outcomes.

Imaging surveillance in high-risk cancer patients needs to balance the risks of cancer recurrence in a time point-specific manner, based on the mix of clinical and molecular predictors, the costs of both false positive and negative findings, and the availability of effective salvage therapies whose early application is beneficial in the course of disease recurrence. In the era of precision medicine, there is pressing need for post-treatment cancer surveillance to be reconceived through a personalised lens.
